# Planning quality and delivery efficiency of sMLC delivered IMRT treatment of oropharyngeal cancers evaluated by RTOG H‐0022 dosimetric criteria

**DOI:** 10.1120/jacmp.v5i4.2014

**Published:** 2004-11-24

**Authors:** X. Ronald Zhu, Christopher J. Schultz, Michael T. Gillin

**Affiliations:** ^1^ Department of Radiation Oncology Medical College of Wisconsin Milwaukee Wisconsin U.S.A.; ^2^ Present address: Department of Radiation Physics, Box 94 The University of Texas M. D. Anderson Cancer Center 1515 Holcombe Blvd. Houston TX 77030 U.S.A.

**Keywords:** IMRT, head and neck, planning quality, delivery efficiency

## Abstract

The time required to deliver intensity‐modulated radiation therapy (IMRT) treatments can be significantly longer than conventional treatments, especially for the segmented multileaf collimator (sMLC) delivery system with a large record and verification (R&V) overhead. In this work, we evaluate the impact of the number of intensity‐modulated beams (IMBs) and the number of intensity levels (ILs) on the quality and delivery efficiency of IMRT plans, generated by the Corvus planning system for sMLC delivery on a Siemens LINAC with the Lantis R&V system. Detailed studies were performed for three image data sets of previously treated oropharyngeal patients. Treatment plans for patient 1 were developed using 5, 7, 9, or 15 evenly spaced axial IMBs as well as one with 7 axial IMBs whose directions were user‐selected, each using ILs of 3, 5, 10, or 20. For patients 2 and 3, plans with 15 IMBs and 20 ILs were not attempted. A total of 42 plans were developed using three oropharyngeal cancer CT image data sets. Plan quality was evaluated by assessing compliance with the Radiation Therapy Oncology Group (RTOG) H‐0022 protocol criteria and the physician's clinical judgment. Plan efficiency was accessed by the number of segments of each plan. We found that for our treatment‐planning and delivery system, an IMRT plan that uses a moderate number of IMBs and ILs, such as 7 or 9 IMBs with 3 or 5 ILs, would appear to be the optimal approach when both quality of the plan and delivery efficiency are considered. Based on this study, we have routinely used 9 IMBs with 3 ILs or 7 IMBs with 5 ILs for head and neck patients. A retrospective comparison indicates that delivery efficiency is improved on the order of 30% compared to plans generated with 9 IMBs with 5 ILs.

PACS number: 87.53.Tf

## I. INTRODUCTION

In recent years, there has been great interest in implementing intensity‐modulated radiation therapy (IMRT) in external beam radiation therapy. IMRT employs nonuniform beam intensity to deliver highly conformal radiation to the targets while minimizing doses to normal tissues and critical organs.^(^ ^1^ ^,^ ^2^ ^)^ Head and neck cancer is one of the attractive sites for IMRT because of the complexity of the anatomy in this region, with many critical and radiation‐sensitive tissues in close proximity to the targeted tumor.^(^ ^3^ ^)^ Recently, the Radiation Therapy Oncology Group (RTOG) activated the first IMRT protocol for phase I/II study of oropharyngeal cancer, H‐0022.^(^ ^4^ ^)^ Conventional radiation therapy for advanced oropharyngeal tumors typically delivers high dose to the major salivary glands (parotid, submandibular, and sublingual) bilaterally. In most cases, this causes a marked reduction in oral saliva output. Xerostomia is the most prevalent late side effect of radiation for head and neck malignancies and is cited by patients as the major cause of decreased quality of life. It has been demonstrated that, using conformal radiation techniques including IMRT, it is feasible to provide adequate irradiation of the targets while sparing major salivary glands.^(^ ^4^ ^)^ To date, no uniform dose‐volume criteria have been universally adopted for the delivery of IMRT for head and neck cancers. Such criteria have been proposed and are currently being tested in the prospective head and neck IMRT trial, RTOG H‐0022. These criteria include stringent requirements for dose coverage of the planning target volumes (PTVs) and for dose limits to critical structures.

Several techniques are available for delivering IMRT treatments, including segmented multileaf collimator (sMLC) and dynamic multileaf collimator (dMLC) intensity modulation.^(^ ^5^ ^)^ The time that is required to deliver IMRT treatments can be significantly longer than for conventional treatments, especially for an sMLC delivery system with a large overhead of the recording and verification (R&V). The prolonged delivery time may result in undesirable consequences, including the increased possibility of intra‐fraction motion, potential radiobiological impact,^(^ ^6^ ^,^ ^7^ ^)^ and schedule conflicts, particularly for busy clinics where treatment machines are already at or near the maximum daily workload. Therefore, it is practically important to develop IMRT plans that can be delivered as efficiently as possible while meeting the stringent dosimetric requirements.

Both treatment plan quality and delivery efficiency depend on the number of intensity‐modulated beams (IMBs) and the number of intensity levels (ILs) for an sMLC delivery system. In general, the higher the number of IMBs and ILs, the better quality the plan and the longer it takes to deliver. Plan quality and delivery efficiency are two requirements that often work against each other. Many groups have researched the topics related to the optimization of IMRT treatment plans, including number of beams,^(^ ^8^ ^–^ ^10^ ^)^ beam angle selection,^(^ ^10^ ^–^ ^12^ ^)^ optimization of beam angles,^(^ ^13^ ^–^ ^18^ ^)^ leaf‐sequencing algorithms,^(^ ^19^ ^,^ ^20^ ^)^ and empirical methods.^(^ ^21^ ^)^ It is generally accepted that results close to optimum can be achieved using fewer than 10 IMBs.^(^ ^22^ ^)^ However, this varies from site to site. For some complex cases, such as head and neck, beam orientation optimization even with 9 beams can be beneficial.^(^ ^14^ ^)^ While they are promising, beam angle optimization and more efficient leaf‐sequencing algorithms are not readily available in common clinical practice. Planners still empirically decide number of beams and orientation of each beam, guided by the above‐mentioned research results and experience of the planner. While most IMRT planning methods assume a continuous modulation of the intensity, it has been shown that good results can be achieved with stepped dose profiles.^(^ ^22^ ^)^ In fact, it was reported that a moderate number of stairsteps with 5 to 7 ILs could result in plans almost as good as those with continuous modulation.^(^ ^23^ ^)^ However, even with 9 IMBs and 5 ILs, some head and neck IMRT plans still require delivery time longer than 30 min for certain combinations of IMRT planning and delivery systems. The purpose of this work is to evaluate the possibility of further reducing the number of ILs and/or IMBs to improve treatment delivery efficiency while maintaining compliance with the RTOG H‐0022 dosimetric criteria for oropharyngeal IMRT treatment. In this study, all IMRT plans were generated by the Corvus planning system for sMLC delivery on a Siemens LINAC with Lantis R&V.

## II. MATERIALS AND METHODS

### A. Target volumes and normal structures

Three CT image data sets of oropharyngeal (two left tonsil, one right tonsil) cancer patients, who were previously treated with IMRT, were used for this study; see Table 1 for the summary of the patient information.

**Table 1:** Patient information summary

Patient	Site	PTV‐66 (cm^3^)	PTV‐60 (cm^3^)	PTV‐54 (cm^3^)	RT Parotid (cm^3^)	LT Parotid (cm^3^)
1	left tonsil	97	—	417	27.3	21
2	right tonsil	75	185	326	25	20.9
3	left tonsil	160	131	88	12.8	10.5

#### A.1 Target volumes

**Figure 1:**
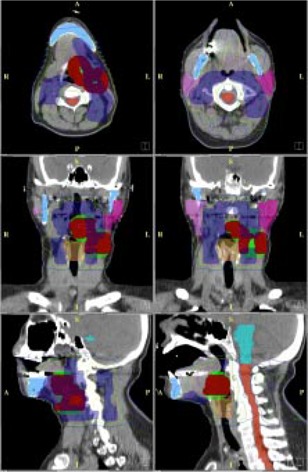
Images for patient 1 in two axial planes, two coronal planes, and two sagittal planes. PTV‐66 (green and red), PTV‐54 (blue), right and left parotids (pink), spinal cord (yellow), brain stem (cyan), and larynx (light brown). Also overlain on the images are isodose lines for the plan with 9 IMBs and 3 ILs; 72.6 Gy (red), 66 Gy (magenta), 54 Gy (yellow), 45 Gy (green), and 30 Gy (blue).

All target volumes were defined according to RTOG H‐0022 protocol. The gross target volume (GTV) included gross disease (tonsil) and palpable lymph nodes in the neck. The clinical target volume (CTV) for the GTV, CTV‐66, was equal to GTV plus at least 5‐mm margins. The planning target volume (PTV) for the CTV‐66, PTV‐66, was created with CTV‐66 plus 5‐mm margins to account for the setup uncertainties. The secondary target is the CTV of lymph node groups or surgical neck levels at risk of subclinical disease, CTV‐54. CTV‐54 defined for image data sets includes the right second, third, and fifth echelon nodes, the left second, third, and fifth echelon modes, retropharyngeal nodes, and submandibular nodes (level 1B). The PTV for the CTV‐54, PTV‐54, was created with CTV‐54 plus 5‐mm margins. RTOG H‐0022 also allows an optional target volume (CTV‐60) to be defined at the discretion of the treating physician. CTV‐60 was defined for patients 2 and 3 (Table 1). An example of target volumes and normal structures (patient 1) is shown in Fig. 1.

#### A. 2 Critical normal structures

The defined critical normal structures include the spinal cord, parotid salivary glands, mandible, brainstem, glottic larynx, submandibular salivary glands, and skin surface. The spinal cord is expanded by a 5‐mm margin to create a planning organ at risk (POAR) for this structure. The tissue within the skin surface and outside all other critical normal structures and PTVs is defined as nontarget tissue.

The CT image data set for patient 1 was used for the “dry run” for the RTOG H‐0022 protocol for our institution. All the contours defined, including both targets and normal structures, were reviewed and approved by the study chair of the protocol as well as the attending radiation oncologist.

### B. Treatment planning

The treatment‐planning software used in this work was Corvus (v.5, NOMOS Corporation, Swickley, PA). The input prescription data required by the Corvus treatment planning included the following: (1) 4‐point prescription dose‐volume histogram (DVH); (2) uncertainties for setup and targets/structures definition (for PTV generation); and (3) treatment unit, beam energy, number of beams, beam directions, and number of level of intensity modulations. The PTVs were assigned a dose goal, a percent volume that may be less than the goal dose, as well as minimum and maximum doses to be delivered. Each normal structure was assigned a dose limit, a percent volume that may receive more than the limit, as well as minimum and maximum doses. A simulated annealing optimization algorithm with the “Continuous Annealer” option was used in this work. This option was chosen because it gives the best resultant dose distributions for plans with complex prescriptions, according to the vendor.^(^ ^24^ ^)^ All plans were done with inhomogeneity correction turned on during optimization and dose calculation. The initial plan for each image data set was executed using 9 IMBs and 5 ILs because it was the standard technique at that time in our clinic.

Treatment plans were developed using 5, 7, 9, and 15 evenly spaced and planner‐selected 7 IMBs using 3, 5, 10, or 20 ILs for patient 1. For 15 IMBs, the plans with 10 and 20 ILs could not be generated because the planning system requires fraction monitor units, but Siemens LINACs do not support it. For patients 2 and 3, plans were developed using 5, 7, and 9 evenly spaced and planner‐selected 7 IMBs using 3, 5, or 10 ILs. A total of 42 plans were developed using the three oropharyngeal cancer CT image data sets. The dosimetric criteria from RTOG H‐0022 used include the following:

1. The prescription dose of 66 Gy (2.2Gy×30fractions) encompasses at least 95% of the PTV of the gross target (PTV‐66), no more than 20% (less than 25% for minor violation) of the PTV‐66 receives greater than 110% of the prescribed dose (72.6 Gy), and no more than 1% of the PTV‐66 receives less than 93% of the prescribed dose (61.4 Gy).
2. The prescription dose of 54 Gy (1.8Gy×30fractions) encompasses at least 95% of the PTV of the neck lymph nodes (PTV‐54), and no more than 20% of the PTV‐54 receives greater than 110% of the prescribed dose to PTV‐54 (59.4 Gy). For minor variation, the 47 Gy isodose surface covers no less than 99% of the PTV‐54, the 54 Gy isodose surface covers no less than 90% of the PTV‐54, and the 72.6 Gy isodose surface (110% of the PTV66 prescription dose) covers no more than 20% of the PTV‐54.
3. Dose to the spinal cord plus a 5‐mm margin is less than 45 Gy.
4. The mean dose to either period less than 26 Gy or at least 50% of the either parotid gland receives less than 30 Gy, or at least 20 cm^3^ of the combined volume of both parotid glands receives less than 20 Gy.

The plans were generated for each image data set using the same optimization constraints but different numbers of IMB and IL. All plans were normalized so that 95% of the PTV‐66 received at least 66 Gy.

### C. Plan evaluation

The treatment plan quality was determined by assessing compliance with RTOG protocol H‐0022 criteria. For plans meeting the dosimetric criteria of H‐0022, the plans with more homogenous dose to the targets and lower doses to the structures were considered to have better quality. When DVH is used to assess the dose homogeneity to the target, the plans with larger percent of PTV receiving prescription dose and smaller percent of volume exceeding the limit dose (e.g., 110% of the prescription dose) were considered to have better quality. In an ideal situation, the DVH plots for target volumes should be a step function, 100% of volume receiving the prescription dose and no volume receiving doses greater than the prescription dose. In practice, this ideal DVH does not exist.

The treatment plan efficiency was accessed by number of segments of each plan. For the LINACs and R&V system used in this work, the average delivery time of each segment, not including the patient setup time, is approximately 0.2 min. Delivery time can be very quickly estimated based on the number of segments for each plan.

### D. Treatment unit

All IMRT plans were generated for delivery on a Siemens digital Mevatron (Primus) with an sMLC and Lantis R&V using 6 MV X‐rays. The dual‐focused MLC consists of 29 pairs leaves, the inner 27 pairs projecting to 1.0‐cm width at isocenter.^(^ ^25^ ^)^

## III. RESULTS AND DISCUSSION

RTOG H‐0022 for oropharyngeal cancer is the first multi‐institutional prospective IMRT study for head and neck cancer. The prescription dose is 66 Gy (2.2Gy×30fractions) to PTV‐66, 54 Gy (1.8Gy×30fractions) to PTV‐54, and optionally 60 Gy (2.0Gy×30fractions) to the PTV‐60. The IMRT plans were initially conceived and designed to be delivered as a “simultaneous integrated boost.”^(^ ^8^ ^)^ Shown in Fig. 1 are images for patient 1 in axial, coronal, and sagittal planes with PTVs and normal structures. Also included in Fig. 1 are isodose lines for the plan generated with 9 IMBs and 3 ILs.

### A. PTV‐66

**Figure 2:**
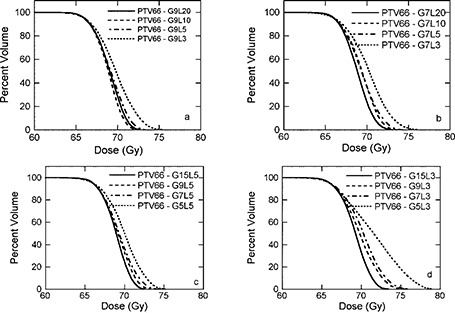
Dose‐volume histograms for PTV‐66 of patient 1. (a) 9 intensity modulated beams (IMBs) with 3, 5, 10, and 20 intensity levels (ILs); (b) 7 IMBs with 3, 5, 10, and 20 ILs; (c) 5 ILs with 5, 7, 9, and 15 IMBs; (d) 3 ILs with 5, 7, 9, and 15 IMBs.

Figure 2 shows DVHs for PTV‐66 of patient 1 for plans with various IMBs and ILs. It should be emphasized that for comparison purposes, all plans were normalized such that 95% of the PTV‐66 received the prescription dose, 66 Gy. In general, the more ILs, the better the plan, although there is very little difference between plans with 5, 10, and 20 ILs for 9 IMBs. There are exceptions to the observation for this particular image data set with the treatment‐planning software (Corvus v.5). For example, as shown in (Fig. 2a), the plan with 9 IMBs and 20 ILs is not better than one with 10 ILs in terms of percent of volume exceeding the 110% of the prescription dose (72.6 Gy). This is an interesting observation and requires further investigation. The plans with 3 ILs for both 7 and 9 IMBs are not as good as plans with larger numbers of ILs in terms of percent of volume exceeding the 110% of the prescription dose (72.6 Gy). But both plans meet the dosimetric criteria of RTOG H‐0022 for PTV‐66 (i.e., less than 20% of the PTV‐66 can receive 110% of the prescription dose) because the volume exceeding 72.6 Gy is 14.6% and 10.8%, respectively, for 7 and 9 IMBs, as listed in Table 2. In fact, for patient 1, all plans meet the dosimetric criteria for PTV‐66, except the plan with 5 IMBs and 3 ILs. This plan fails because 40% of the PTV‐66 received a dose of 72.6 Gy or more. For a given number of ILs, the larger number of IMBs, the better the plan, as shown in (Figs. 2c) and (d). The plans with 5 IMBs are clearly the worst in terms of DVH for PTV‐66. There is another dosimetric requirement for PTVs, namely, no more than 1% of any PTV will receive less than 93% of its prescribed dose. For PTV‐66, the 93% of the prescribed dose is 61.4 Gy. All plans generated this study meet this particular requirement. Similar results are observed for patients 2 and 3 (see Tables 2B and C).

**Table 2A:** Patient 1 DVH summary of PTV‐66 for 5, 7, 9, and 15 intensity modulated beams (IMBs) IMRT plans with 3, 5, 10, and 20 intensity levels (ILs)

	5 IMBs		7 IMBs*		9 IMBs		15 IMBs**	
IL	%Vol≥72.6Gy	Max dose (Gy)	%Vol≥72.6Gy	Max dose (Gy)	%Vol≥72.6Gy	Max dose (Gy)	%Vol≥72.6Gy	Max dose (Gy)
3	40	80.3	14.6/11.1	77.9/76.5	10.8	76.4	1.2	74.2
5	11.7	76.7	4.6/1.1	75.2/74.2	1.5	74.9	0.2	73.4
10	7.5	75.5	2.0/1.3	75.8/73.5	0.1	73.2	—	—
20	4.2	74.8	0.6/0.5	74.7/73.5	0.3	73.7	—	—

B. PTV‐60

**Figure 3:**
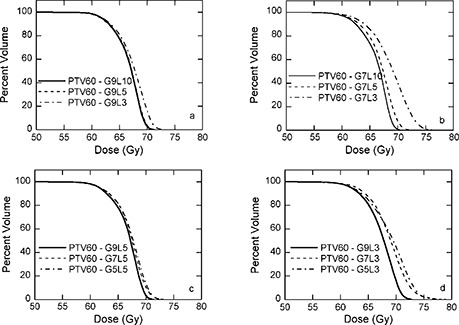
Dose‐volume histograms for PTV‐60 of patient 2. (a) 9 IMBS with 3, 5, and 10 ILs; (b) 7 IMBs with 3, 5, and 10 ILs; (c) 5 ILs with 5, 7, and 9 IMBs; (d) 3 ILs with 5, 7, and 9 IMBs.

DVHs for PTV‐60 of patient 2 are displayed in Fig. 3. Again, for a given number of IMBs, the more ILs, the better the plan; for a given number of ILs, the more IMBs, the better plan. Similar results are observed for patient 3.

**Figure 4:**
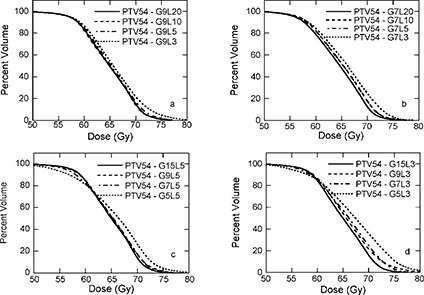
Dose‐volume histograms for PTV‐54 of patient 1. (a) 9 intensity modulated beams (IMBs) with 3, 5, 10, and 20 intensity levels (ILs); (b) 7 IMBs with 3, 5, 10, and 20 ILs; (c) 5 ILs with 5, 7, 9, and 15 IMBs; (d) 3 ILs with 5, 7, 9, and 15 IMBs.

Fig. 3. Dose‐volume histograms for PTV‐60 of patient 2. (a) 9 IMBs with 3, 5, and 10 ILs; (b) 7 IMBs with 3, 5, and 10 ILs; (c) 5 ILs with 5, 7, and 9 IMBs; (d) 3 ILs with 5, 7, and 9 IMBs.

C. PTV‐54

Figure 4 displays DVHs for PTV‐54 of patient 1 for plans with various IMBs and ILs. The dosimetric criterion for PTV‐54, at least 95% of PTV‐54 receives prescription dose, 54 Gy, and less than 20% of PTV‐54 receives of 110% of the prescription dose, 59.4 Gy, is very difficult to meet. In fact, for each plan generated for patient 1, more than 80% of the PTV‐54 received at least 59.4 Gy, which is significantly larger than 20%. The reason for this is that PTV‐54 is in close proximity to the critical normal structures, such as the salivary glands and the spinal cord. To achieve the sharp dose drop‐off at the critical normal structures, dose inhomogeneity in the PTV is larger. Recognizing this difficulty, RTOG H‐0022 allows minor variation for PTV‐54. The criteria for such a minor protocol variation are as follows: 47 Gy isodose surface covers no less than 99% of the PTV‐54, and the 54 Gy isodose surface covers no less than 90% of the PTV‐54; the 72.6 Gy isodose surface (110% of PTV‐66 prescription dose) covers no more than 20% of the PTV‐54. All plans generated can meet the criteria for PTV‐54 with minor variation, as shown in Fig. 4 and Table 3.

**Figure 5:**
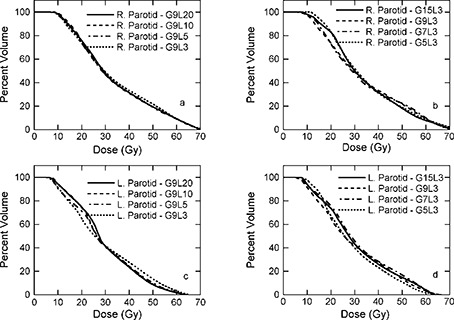
Dose‐volume histograms for parotid glands of patient 1. (a) 9 intensity modulated beams (IMBs) with 3, 5, 10, and 20 intensity levels (ILs) for the right parotid gland; (b) 3 ILs with 5, 7, 9, and 15 IMBs for the right parotid gland; (c) 9 IMBs with 3, 5, 10, and 20 ILs for the left parotid gland; (d) 3 ILs with 5, 7, 9, and 15 IMBs for the left parotid gland.

**Table 2B:** Patient 2 DVH summary of PTV‐66 for 5, 7, and 9 IMBs IMRT plans with 3, 5, and 10 ILs

	5 IMBs		7 IMBs*		9 IMBs	
IL	%Vol≥72.6Gy	Max dose (Gy)	%Vol≥72.6Gy	Max dose (Gy)	%Vol≥72.6Gy	Max dose (Gy)
3	24.0	77.3	7.5/4.6	77.2/75.7	0.2	73.4
5	0.1	72.9	<0.1/0.2	72.6/73.4	0.0	71.8
10	<0.1	73.6	0.0/0.0	71.9/72.3	0.0	71.2

### D. Normal tissues

**Figure 6:**
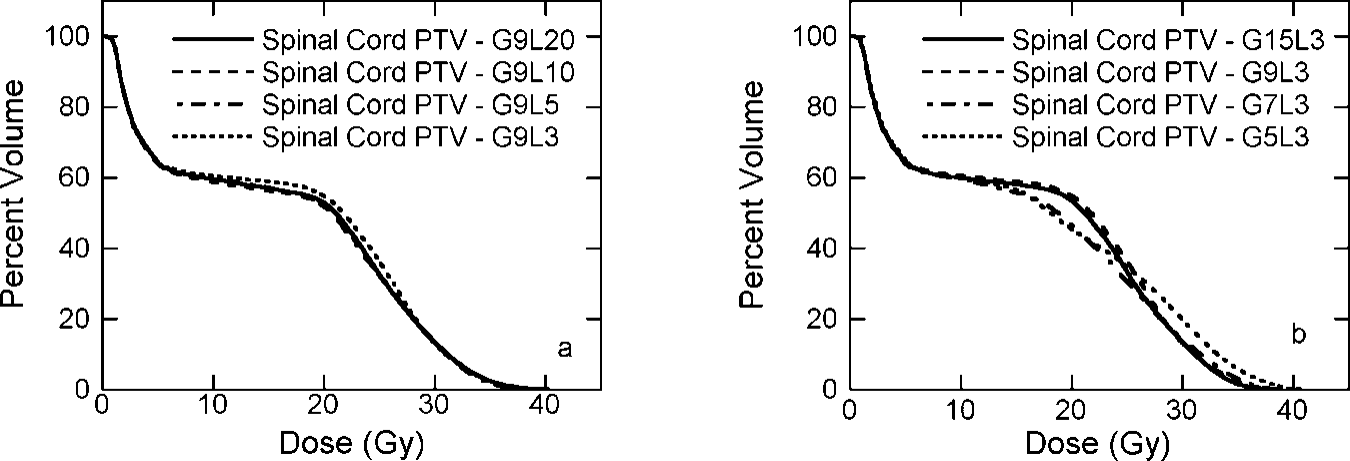
Dose‐volume histograms for planning organ at risk (POAR) of the spinal cord of patient 1. (a) 9 IMBs with 3, 5, 10, and 20 ILs; (b) 3 ILs with 5, 7, 9, and 15 IMBs.

Shown in Fig. 5 are DVHs for the parotid salivary glands of patient 1. The dosimetric criteria for parotid glands are as follows: (1) the mean dose to either parotid less than 26 Gy or (2) at least 50% of the either parotid gland receive less than 30 Gy or (3) at least 20 cm^3^ of the combined volume of both parotid glands receive less than 20 Gy. All plans meet requirement (2), as listed in Tables 4 and 5. Displayed in Fig. 6 are DVHs for the POAR of the spinal cord of patient 1. The dosimetric criterion is the maximum dose less than 45 Gy. Figure 6 clearly demonstrates that all plans meet this requirement. Similar results are observed for spinal cord of patients 2 and 3.

**Figure 7:**
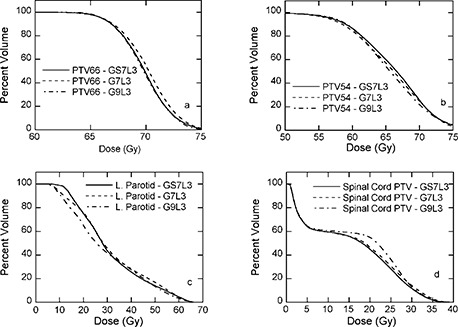
Dose‐volume histograms for plans with 7 user‐selected (solid line), 7 (dashed line), and 9 (dashed‐dotted line) evenly spaced IMBs with 3 ILs of patient 1. (a) PTV‐66; (b) PTV‐54; (c) left parotid gland; (d) POAR of the spinal cord.

**Table 2C:** Patient 3 DVH summary of PTV‐66 for 5, 7, and 9 IMBs IMRT plans with 3, 5, and 10 ILs

	5 IMBs		7 IMBs*		9 IMBs	
IL	%Vol≥72.6Gy	Max dose (Gy)	%Vol≥72.6Gy	Max dose (Gy)	%Vol≥72.6Gy	Max dose (Gy)
3	37.2	80.8	20.8/21.0	80.6/79.6	10.1	76.1
5	21.9	77.6	7.3/8.1	77.1/76.7	3.1	75.1
10	10.5	76.9	2.6/3.9	75.2/75.4	0.8	75.3

* For 7 IMBs, the numbers in the front of slash are for evenly spaced 7 beams, and the numbers after the slash are for planner‐selected 7 beams.

** For 15 IMBs, the plans with 10 and 20 ILs could not be generated due to the limitation of the combined delivery system and treatment‐planning system.

**Table 3A:** Patient 1 DVH summary of PTV‐54 for 5, 7, 9, and 15 IMB IMRT plans with 3, 5, 10, and 20 ILs

	5 IMBs			7 IMBs*			9 IMBs			15 IMBs**		
IL	%Vol≥47Gy	%Vol≥54Gy	%Vol≥72.6Gy	%Vol≥47Gy	%Vol≥54Gy	% Vol ≤72.6Gy	%Vol≥47Gy	%Vol≥54Gy	%Vol≥72.6Gy	%Vol≥47Gy	%Vol≥54Gy	%Vol≥72.6Gy
3	100.0	95.8	21.7	100.0/100.0	97.8/98.0	10.7/10.7	100	95.3	10.3	100.0	98.3	4.0
5	100.0	95.0	11.4	100.0/100.0	97.6/97.9	4.8/6.6	100	98.0	5.9	100.0	98.4	3.5
10	100.0	95.5	9.4	100.0/100.0	97.9/97.9	4.8/5.5	100	98.1	3.7	—	—	—
20	100.0	95.3	7.6	100.0/100.0	97.0/97.7	2.9/4.7	100	98.1	4.0	—	—	—

**Table 3B:** Patient 2 DVH summary of PTV‐54 for 5, 7, and 9 IMBs IMRT plans with 3, 5, and 10 ILs

	5 IMBs			7 IMBs*			9 IMBs		
IL	%Vol≥47Gy	%Vol≥54Gy	%Vol≥72.6Gy	%Vol≥47Gy	%Vol≥54Gy	%Vol≥72.6Gy	%Vol≥47Gy	%Vol≥54Gy	%Vol≥72.6Gy
3	99.8	92.9	4.8	99.9/99.9	95.9/94	2.9/0.6	99.9	96.5	0.3
5	99.8	91.8	0.4	99.9/99.8	94.1/90.7	0.2/0.1	99.9	95.2	0.0
10	99.9	93.3	<0.1	99.9/99.9	95.6/90.8	<0.1/0.0	99.9	96.6	0.0

**Table 3C:** Patient 3 DVH summary of PTV‐54 for 5, 7, and 9 IMBs IMRT plans with 3, 5, and 10 ILs

	5 IMBs			7 IMBs*			9 IMBs		
IL	% Vol ≥47 Gy	%Vol≥54Gy	% Vol ≥72.6 Gy	% Vol ≥≥47 Gy	% Vol ≥54 Gy	% Vol ≥72.6 Gy	% Vol ≥47 Gy	% Vol ≥54 Gy	% Vol ≥72.6 Gy
3	99.9	94.6	2.6	100/100	98.6/97.9	1.3/0.9	100	97.6	0.3
5	99.8	93.8	2.0	100/100	96.4/95.0	0.2/<0.1	100	97.0	0.2
10	99.8	93.6	0.5	100/100	96.4/95.9	<0.1/<0.1	100	97.6	0.0

* For 7 IMBs, the numbers in the front of slash are for evenly spaced 7 beams, and the numbers after the slash are for planner‐selected 7 beams.

** For 15 IMBs, the plans with 10 and 20 ILs could not be generated due to the limitation of the combined delivery system and treatment‐planning system.

**Table 4A:** Patient 1 DVH summary of right parotid for 5, 7, 9, and 15 IMBs IMRT plans with 3, 5, 10, and 20 ILs.

	5 IMBs	7 IMBs*	9 IMBs	15 IMBs**
IL	%Vol≥30Gy	%Vol≥30Gy	%Vol≥30Gy	%Vol≥30Gy
3	52.0	47.5/50.0	49.0	49.9
5	50.1	48.5/49.2	46.8	46.9
10	47.0	48.2/49.5	46.5	—
20	50.1	48.6/50.0	47.8	—

**Table 4B:** Patient 2 DVH summary of right parotid for 5, 7, and 9 IMBs IMRT plans with 3, 5, and 10 ILs

	5 IMBs	7 IMBs*	9 IMBs
IL	%Vol≥30Gy	%Vol≥30Gy	%Vol≥30Gy
3	43.9	48.0/35.3	46.5
5	49.0	49.5/39.3	46.2
10	48.5	46.4/38.2	46.2

**Table 4C:** Patient 3 DVH summary of right parotid for 5, 7, and 9 IMBs IMRT plans with 3, 5, and 10 ILs

	5 IMBs	7 IMBs*	9 IMBs
IL	%Vol≥30Gy	%Vol≥30Gy	%Vol≥30Gy
3	44.0	36.4/29.6	44.9
5	46.0	30.5/21.2	39.0
10	46.4	43.6/26.9	38.2

* For 7 IMBs, the numbers in the front of slash are for evenly spaced 7 beams, and the numbers after the slash are for planner‐selected 7 beams.

** For 15 IMBs, the plans with 10 and 20 ILs could not be generated due to the limitation of the combined delivery system and treatment‐planning system.

**Table 5A:** Patient 1 DVH summary of left parotid for 5, 7, 9, and 15 IMBs IMRT plans with 3, 5, 10, and 20 ILs

	5 IMBs	7 IMBs*	9 IMBs	15 IMBs**
IL	%Vol≥30Gy	%Vol≥30Gy	%Vol≥30Gy	%Vol≥30Gy
3	40.0	46.0/43.5	41.8	43.0
5	39.0	41.8/40.1	40.7	41.5
10	39.9	44.9/42.9	41.2	—
20	40.5	43.0/41.9	41.3	—

**Table 5B:** Patient 2 DVH summary of left parotid for 5, 7, and 9 IMBs IMRT plans with 3, 5, and 10 Ils

	5 IMBs	7 IMBs*	9 IMBs
IL	%Vol≥30Gy	%Vol≥30Gy	%Vol≥30Gy
3	44.8	40.6/37.7	39.6
5	39.7	41.4/36.5	37.5
10	39.9	42.9/35.0	39.5

**Table 5C:** Patient 3 DVH summary of left parotid for 5, 7, and 9 IMBs IMRT plans with 3, 5, and 10 ILs

	5 IMBs	7 IMBs*	9 IMBs
IL	%Vol≥30Gy	%Vol≥30Gy	%Vol≥30Gy
3	55.5	54.1/62.3	55.6
5	61.2	52.4/57.2	64.9
10	59.7	57.4/53.6	59.4

* For 7 IMBs, the numbers in the front of slash are for evenly spaced 7 beams, and the numbers after the slash are for planner‐selected 7 beams.

** For 15 IMBs, the plans with 10 and 20 ILs could not be generated due to the limitation of the combined delivery system and treatment‐planning system.

RTOG H‐0022 requires that no more than 1% or 1 cm^3^ of the tissue outside the PTVs receives more than 72.6 Gy (110% of the prescribed dose to PTV‐66). Table 6 lists the dose and volume parameters for nontarget tissue. Each plan meets this requirement because each has less than 1%, but more than 1 cm^3^, of the tissue exceeding 72.6 Gy. Other critical structures include glottic larynx (2/3 below 50 Gy), brainstem (54 Gy), and mandible (70 Gy). The protocol encourages the participants to remain within these limits. However, some plans have a significant volume of nontarget tissue (still within 1%) receiving a dose greater than 72.6 Gy, despite meeting all the dosimetric criteria. For example, the plan for patient 1 with 7 IMBs and 3 ILs may be clinically unacceptable due to the larger volume $\left(43{\text{mm}}^{3}\right)$ of nontarget tissue receiving a dose greater than 72.6 Gy.

**Table 6A:** Patient 1 DVH summary of nontarget tissue for 5, 7, 9, and 15 IMBs IMRT plans with 3, 5, 10, and 20 ILs

	5 IMBs			7 IMBs*			9 IMBs			15 IMBs**		
IL	$\text{Vol}({\text{cm}}^{3})\text{IL}\geq 72.6\text{Gy}$	%Vol≤72.6Gy	Max dose (Gy)	$\text{Vol}({\text{cm}}^{3})\geq 72.6\;\text{Gy}$	%Vol≤72.6Gy	Max dose (Gy)	$\text{Vol}({\text{cm}}^{3})\geq 72.6\;\text{Gy}$	%Vol≥72.6Gy	Max dose (Gy)	$\text{Vol}({\text{cm}}^{3})\leq 72.6\text{Gy}$	%Vol≥72.6Gy	Max dose (Gy)
3	70	0.8	86.9	43/24.4	0.5/0.3	82.0/83.3	26	0.3	82.7	6.2	0.1	77.4
5	31.8	0.4	85.2	21.5/13.2	0.3/0.2	81.2/79.0	12.3	0.1	78.9	5.0	0.1	81.5
10	30.3	0.4	82.9	26.4/7.0	0.3/0.1	81.1/80.0	5.9	0.1	78.3	—	—	—
20	28.5	0.3	82.2	20.3/9.8	0.3/0.1	81.2/77.9	4.5	0.1	76.8	—	—	—

**Table 6B:** Patient 2 DVH summary of nontarget tissue for 5, 7, and 9 IMBs IMRT plans with 3, 5, and 10 ILs

	5 IMBs			7 IMBs*			9 IMBs		
IL	$\text{Vol}({\text{cm}}^{3})\leq 72.6\text{Gy}$	%Vol≤72.6Gy	Max dose (Gy)	$\text{Vol}({\text{cm}}^{3})\leq 72.6\text{Gy}$	%Vol≤72.6Gy	Max dose (Gy)	$\text{Vol}({\text{cm}}^{3})\leq 72.6\text{Gy}$	%Vol≤72.6Gy	Max dose (Gy)
3	12.4	0.4	81.2	7.8/3.4	0.2/0.1	79.1/76.5	0.3	<0.1	74.5
5	0.6	<0.1	79.2	0.5/0.6	<0.1/<0.1	74.5/74.9	0.0	0.0	72.1
10	0.3	<0.1	77.5	<0.1/0.1	0.0/0.0	74.1/73.4	0.0	0.0	71.9

**Table 6C:** Patient 3 DVH summary of nontarget tissue for 5, 7, and 9 IMBs IMRT plans with 3, 5, and 10 ILs

	5 IMBs			7 IMBs*			9 IMBs		
IL	$\text{Vol}({\text{cm}}^{3})\geq 72.6\;\text{Gy}$	%Vol≥72.6Gy	Max dose (Gy)	$\text{Vol}({\text{cm}}^{3})\geq 72.6\;\text{Gy}$	%Vol≥72.6Gy	Max dose (Gy)	$\text{Vol}({\text{cm}}^{3})\geq 72.6\;\text{Gy}$	%Vol≥72.6Gy	Max dose (Gy)
3	29.5	0.9	87.9	24.2/21.6	0.8/0.7	80.6/81.3	7.7/	0.3	80.5
5	13.9	0.5	77.6	4.2/5.8	0.1/0.2	75.9/77.4	2.1/	<0.1	74.7
10	5.6	0.2	76.9	1.4/2.0	≥0.1/≥0.1	74.8/77.7	0.6	≥0.1	74.6

* For 7 IMBs, the numbers in the front of slash are for evenly spaced 7 beams, and the numbers after the slash are for planner‐selected 7 beams.

** For 15 IMBs, the plans with 10 and 20 ILs could not be generated due to the limitation of the combined delivery system and treatment‐planning system.

### E. User‐selected beam angle

The above discussion is focused on plans generated with IMBs with equally spaced gantry angles. It has been reported that for 9 IMBs and more, gantry angle selection may be less important,^(^ ^8^ ^)^ while for other cases gantry angle selection may still be beneficial.^(^ ^14^ ^)^ We therefore also tested user‐selected beam angles using 7 IMBs. The general angle selection method was to use a beam's‐eye view planning tool to maximize the target exposure and minimize normal structures exposure while avoiding parallel‐opposed beams. Shown in Fig. 7 are DVHs for the plan generated by 7 user‐selected IMBs compared with plans for 7 and 9 evenly spaced IMBs with 3 ILs. The plan with 7 user‐selected IMBs is nearly identical to the plan with 9 evenly spaced beams and outperformed the plan with 7 evenly spaced beam in terms of PTV‐66, as shown in (Fig. 7a). Similar DVHs are obtained for PTV‐54, left parotid, and spinal cord between these three plans, as illustrated in (Figs. 7b) to (d). For patient 2, there is at least a 5% reduction in volume of each parotid gland receiving at least 30 Gy for the user‐selected 7 beam plans compared with plans generated with 7 evenly spaced beams. For patient 3, only the right parotid gland shows significant (at least 5%) reduction (see Tables 4 and 5). This result demonstrates that for number of beams fewer than 9, beam angle selection and optimization could be important, which is consistent with the literature.^(^ ^8^ ^,^ ^14^ ^)^

**Figure 8:**
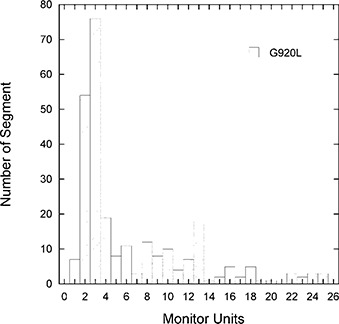
Monitor units histogram for the plan with 9 IMBs and 20 ILs for patient 1.

### F. Treatment efficiency

Treatment efficiency is governed by the number of segments for the delivery as well as the R&V and system used in this work. Table 7 shows a number of segments for each plan. It is obvious that the plans with the least number of IMBs and ILs has the fewest segments and therefore is the most efficient one to deliver. However, for patient 1, it is also the plan that fails to meet the dosimetric criteria of the H‐0022 protocol and has the largest volume of the nontarget tissue receiving a dose greater than 72.6 Gy. The plans with 7 or 9 IMBs and 3 or 5 ILs may be the optimal choices when both plan quality and delivery efficiency are considered. When number of beams used is fewer than 9, user‐selected beam angles could be helpful for improving the quality of plan, while keeping the plan delivery efficient.

The delivery times for plans of patient 1 listed in Table 7 are estimated to be 8 min to 55 min, corresponding to 42 to 276 segments using the average value of 0.2 min each segment for our delivery and R&V system. The total delivery time includes beam‐on time, the time needed for the MLC to move to the next shape, R&V system overhead time, and the time needed for the gantry to rotate to the next position. The R&V system has a relatively large overhead for the delivery system studied. For this reason, the total delivery time is best estimated by the number of segments, not the number of monitor units. For a given of number of segments, reducing the time for the other above‐mentioned components would improve treatment efficiency. A significant gain in treatment efficiency could be achieved by reducing the overhead of the R&V system. This would necessarily require the LINAC and R&V system manufacturers to work together to provide such a solution. For the delivery and R&V system studied here, reducing the number of segments is the most effective way of improving delivery efficiency. For example, using the image data set of patient 1, one could select the plan with 7 user‐selected IMBs and 3 ILs. This plan meets the dosimetric criteria of H‐0022 with minor variation yet requires approximately 13 min to be delivered, which is perhaps not significantly longer than some conventional head and neck treatment approaches, particularly when posterior electron and nodal boosting required.

As a result of this study, we have routinely used 9 IMBs with 3 ILs or 5 IMBs with 7 ILs for head and neck cancers, including oropharynx (tonsil, base of tongue, and palate), nasopharynx, unknown primary, pyrimform sinus, and hypopharynx. Reviewing selected head and neck patients treated using 9 IMBs with 5 ILs (8 patients) or 3 ILs (24 patients), we found that, on average, the number of segments is reduced from 143±10 to 101±10 and the number of monitor units from 1983±341 to 1470±247, as shown in Table 8. This represents a reduction of approximately 30% in either the number of segments or the number of monitor units. With this approach, the treatment time in our clinic for these types of patients has been reduced from approximately 30 min to 20 min. Patients included in this comparison were those who had at least one primary target treating to dose 60 Gy to 70 Gy and the bilateral neck nodal regions, including the second, third, and fifth echelon nodes, to 45 Gy to 54 Gy.

**Table 7A:** Number of segments for plans with 5, 7, 9, and 15 IMBs and 3, 5, 10, and 20 ILs for patient 1

ILs	5 IMBs	7 IMBs*	9 IMBs	15 IMBs**
3	42	68/66	94	166
5	65	99/96	127	231
10	96	143/144	197	—
20	129	199/196	276	—

**Table 7B:** Number of segments for plans with 5, 7, and 9 IMBs and 3, 5, and 10 ILs for patient 2

ILs	5 IMBs	7 IMBs	9 IMBs
3	41	62/69	92
5	60	93/92	127
10	90	138/140	180

**Table 7C:** Number of segments for plans with 5, 7, and 9 IMBs and 3, 5, and 10 ILs for patient 3

ILs	5 IMBs	7 IMBs	9 IMBs
3	41	55/57	81
5	58	80/77	103
10	78	112/115	156

* For 7 IMBs, the numbers in the front of slash are for evenly spaced 7 beams, and the numbers after the slash are for planner‐selected 7 beams.

** For 15 IMBs, the plans with 10 and 20 ILs could not be generated due to the limitation of the combined delivery system and treatment‐planning system.

**Table 8:** Comparison number of segments and number of monitor units for head and neck patients, including oropharynx (tonsil, base of tongue, and palate), nasopharynx, unknown primary, pyrimform sinus, and hypopharynx, treated using 9 IMBs with 5 and 3 ILs

	5 ILs	3 ILs	Difference
segments	143±10	101±10	29%
monitor units	1983±341	1470±247	26%

### G. Delivery accuracy

For Siemens LINACs such as the one used in this studied, only integer monitor units can be delivered. as the numbers of beams and intensity levels are increased, the relative dosimetric error on each segment may increase due to the effect of monitor unit linearity. To estimate this error, we have analyzed the plan with 9 IMBs and 20 ILs, which has the most segments for patient 1. The histogram of monitor units for this plan is shown in Fig. 8. It is obvious that there are a significant number of segments with 2 or 3 monitor units. assuming the monitor unit linearity error is about 5% for 1 monitor unit, 2% for 2 to 4 monitor units, and 1% for 5 and more monitor units,^(^ ^26^ ^)^ the largest error is about 1.5% for one of the IMBs and the average error over 9 IMBs is 1.3%. In this work, we attempted to reduce the number of segments to improve delivery efficiency. With fewer segments there are fewer segments with very small monitor units. Therefore the error is smaller. For example, for the plans with 9 IMBs and 3 ILs and 7 IMBs and 5 ILs of patient 1, the minimum monitor units are 12 and 11, respectively. Thus the error is expected to be about 1% or less.

## IV. CONCLUSION

We have performed a detailed comparative treatment‐planning study for three CT image data sets of oropharyngeal cancer patients. Treatment‐planning quality and delivery efficiency for sMLC delivered IMRT treatments are evaluated using RTOG H‐0022 dosimetric criteria. Both number of IMBs and ILs have a significant influence on the quality and efficiency of IMRT treatment using sMLC delivery. A treatment plan that uses a moderate number of IMBs and ILs, such as 7 or 9 IMBs with a 3 or 5 ILs, appears to be the optimal approach when both plan quality and delivery efficiency are considered. It should be emphasized that this study is specifically for the Corvus planning system combined with the sMLC on a Siemens LINAC and R&V system (Lantis). The conclusions drawn from this study should not be applied directly to a different planning, delivery, and R&V system. Even for an identical system, the conclusions should not be used without careful analysis and verification by the treating physician and physicist. Nevertheless, the methodology presented here could be adopted by clinics with similar hardware and software attempting to improve delivery efficiency but cannot access more advanced techniques such as beam angle optimization and efficient leaf‐sequencing algorithms.
